# Tobacco use in people with severe mental illness: Findings from a multi-country survey of mental health institutions in South Asia

**DOI:** 10.18332/tid/174361

**Published:** 2023-12-14

**Authors:** Sukanya Rajan, Alex Mitchell, Gerardo A. Zavala, Danielle Podmore, Humaira Khali, Asiful H. Chowdhury, Krishna Prasad Muliyala, Koralagamage Kavindu Appuhamy, Faiza Aslam, Asad T. Nizami, Rumana Huque, David Shiers, Pratima Murthy, Najma Siddiqi, Kamran Siddiqi, on behalf of the IMPACT research team

**Affiliations:** 1 National Institute of Mental Health and Neurosciences, Bangalore, India; 2 Department of Health Sciences, University of York, York, United Kingdom; 3 Institute of Psychiatry, Rawalpindi, Pakistan; 4 ARK Foundation, Dhaka, Bangladesh; 5 Psychosis Research Unit, Greater Manchester Mental Health National Health Service Foundation Trust, Manchester City, United Kingdom; 6 Division of Psychology and Mental Health, The University of Manchester, Manchester City, United Kingdom; 7 School of Medicine, Keele University, Keele, United Kingdom; 8 Bradford District Care National Health Service Foundation Trust, Bradford, United Kingdom; 9 Hull York Medical School, Hull, United Kingdom; 10 Hull York Medical School, York, United Kingdom

**Keywords:** severe mental illness, smoking, smokeless tobacco, cessation

## Abstract

**INTRODUCTION:**

People with severe mental illness (SMI) tend to die early due to cardiovascular and respiratory diseases, which may be linked to tobacco use. There is limited information on tobacco use in people with SMI in low- and middle-income countries where most tobacco users reside. We present novel data on tobacco use in people with SMI and their access to tobacco cessation advice in South Asia.

**METHODS:**

We conducted a multi-country survey of adults with SMI attending mental health facilities in Bangladesh, India, and Pakistan. Using data collected with a standardized WHO STEPS survey tool, we estimated the prevalence and distribution of tobacco use and assessed receipt of tobacco cessation advice.

**RESULTS:**

We recruited 3874 participants with SMI; 46.8% and 15.0% of men and women consumed tobacco, respectively. Smoking prevalence in men varied by country (Bangladesh 42.8%, India 20.1% and Pakistan 31.7%); <4% of women reported smoking in each country. Smokeless tobacco use in men also varied by country (Bangladesh 16.2%, India 18.2% and Pakistan 40.8%); for women, it was higher in Bangladesh (19.1%), but similar in India (9.9%) and Pakistan (9.1%). Just over a third of tobacco users (38.4%) had received advice to quit tobacco. Among smokers, 29.1% (n=244) made at least one quit attempt in the past year. There was strong evidence for the association between tobacco use and the severity of depression (OR=1.29; 95% CI: 1.12–1.48) and anxiety (OR=1.29; 95% CI: 1.12–1.49).

**CONCLUSIONS:**

As observed in high-income countries, we found higher tobacco use in people with SMI, particularly in men compared with rates reported for the general population in South Asia. Tobacco cessation support within mental health services offers an opportunity to close the gap in life expectancy between SMI and the general population.

**STUDY REGISTRATION:**

ISRCTN88485933; https://doi.org/10.1186/ISRCTN88485933 39

## INTRODUCTION

Severe mental illness (SMI) refers to mental health conditions (e.g. schizophrenia, bipolar disorder) that are often so debilitating that the person’s ability to engage in functional and occupational activities is severely impaired^1^. People with SMI have a life expectancy of 10–15 years lower than the general population^2^. The excess mortality in this population, compared to the general population, is primarily due to a higher prevalence of cardiovascular and respiratory diseases^3^. These chronic physical conditions^4^ may be a consequence of health risk behaviors including the use of tobacco and alcohol, poor diet and physical inactivity in this population^5,6^.

Studies, mostly from high-income countries, suggest that people with SMI smoke more frequently and in greater quantities than the general population, have more severe nicotine dependence, and face worse health outcomes as a result of tobacco use^7,8^. Lack of social support, stigma, poor cognitive function, anxiety, medication side effects and limited self-efficacy are some of the factors that have been associated with the increased burden of tobacco use in this population^9^. Tobacco use has been linked with reduced medication effectiveness, increased mental illness relapse rates and hospitalization in this population^10^. The prevalence of tobacco use in people with SMI has been reported to be as high as 50–90% in some high-income countries (HICs)^7,11^.

Despite a decline in smoking prevalence in the general population, there has been little change seen in smoking trends among those with SMI^8^. Unfortunately, the benefits of smoking cessation programs have not yet been extended to the SMI population^12^. Despite the dramatic reduction in smoking rates in the general population during the past 40 years, there has been almost no reduction in smoking prevalence among people with SMI (this is the case even in HICs).

Over 80% of tobacco users now live in low- and middle-income countries (LMIC)^13^, with access to cheaper, poorly regulated, and diverse range of tobacco products. There is often little awareness of tobacco-related harms and poor access to cessation advice or treatment in these countries^14,15^. While there are population-based surveys in LMICs, there is very limited information on the use of tobacco among those with chronic physical and mental illnesses such as SMI^16,17^. Tobacco use may be contributing to the larger mortality gap between people with SMI and the general population seen in LMICs. A better understanding of patterns of tobacco use in people with SMI is therefore important – these individuals are more likely to be in touch with health services on a regular basis with potentially greater opportunities to receive advice and support for tobacco cessation compared to the general population. The factors responsible for their susceptibility to tobacco use may differ between LMICs and HICs and that knowledge can help in contextualizing smoking cessation interventions.

We examined the prevalence and distribution of tobacco use in people with SMI in three high-tobacco burden South Asian countries. We also assessed the extent to which they received tobacco cessation advice and explored the associations between tobacco use sociodemographic variables, and mental and physical comorbidities.

## METHODS

### Setting and participants

This study utilized data collected as part of a cross-sectional survey of health, health-risk behaviors, and healthcare use in people with SMI in three South Asian countries: the IMPACT study [ISRCTN registry: 88485933]^18,19^. The survey included people aged ≥18 years with a clinician-diagnosed SMI. The diagnoses were confirmed using the international neuropsychiatric interview - MINI version 6.0 and included schizophrenia, schizoaffective disorder, severe depression with psychotic symptoms, and bipolar affective disorder. The participants attended three national institutes of mental health in Bangladesh, India, and Pakistan, between July 2019 and March 2022^18^.

Trained researchers screened the participants to ensure that the inclusion criteria were met, followed by assessing their willingness to participate and obtaining written and informed consent. The details of these have been published elsewhere^18^. Participants without the capacity to consent or who were unwilling to complete the study questionnaire were excluded. The survey was administered in one of the following languages: Bangla, English, Hindi, Kannada, and Urdu.

The study received ethics approval from the ethics committees of the Department of Health Sciences at the University of York (UK), the Centre for Injury Prevention and Research (Bangladesh), the Health Ministry Screening Committee, the Indian Council of Medical Research (India) and National Bioethics Committee (Pakistan).

### Variables

The following variables were used in our analysis: 1) The tobacco module from the World Health Organization STEPwise Approach to Risk Factor Surveillance (STEPS) was used to assess tobacco-related behaviors; 2) Demographic details such as age, sex, education level, marital status, employment, and income using the WHO Stepwise Approach to Surveillance (STEPS) demographic module, and included questions about the age of initiation, the frequency and the amount of tobacco smoked each day; 3) Previous diagnosis of diabetes, heart disease and hypertension, and advice on smoking cessation from a health worker collected by using STEPS instrument V. 3.2; and 4) International neuropsychiatric interview using MINI version 6.0 to confirm the psychiatric diagnosis and to assess the severity of depression and anxiety symptoms we used the Patient Health Questionnaire (PHQ-9) and Generalized Anxiety Symptoms (GAD-7) scales.

### Sample size

The aim was to build as large a sample as possible^18^. As an indicative example of survey precision to address some of the key research questions, a sample size estimate for investigating the prevalence of diabetes was provided in the protocol. Assuming a prevalence estimate of 10%, 857 participants per country were needed to achieve ±2% precision assuming a 95% confidence interval.

### Statistical analysis

Participant characteristics, tobacco use, and receipt of tobacco cessation advice data were summarized descriptively for each country (Bangladesh, India, and Pakistan) and overall. Continuous measures were reported as means and standard deviations (and/or median with interquartile range, and range, as appropriate), Categorical data were reported as frequencies and percentages. The prevalence of current tobacco use (smoked and/or smokeless), past tobacco use (smoked only) and ever tobacco use (current and/or past use) were calculated with 95% confidence intervals for each country, for males and females separately. The prevalence of current tobacco use was also stratified by age, type of SMI, duration of SMI, and income tertile. Similarly, the prevalence of smoking cessation advice was calculated overall and stratified by the above factors. The prevalence of smoking cessation advice was not calculated separately for males and females.

Logistic regression models were used to calculate the unadjusted and adjusted odds ratios of currently using tobacco, and its association with physical (diabetes, heart disease and hypertension) and mental health conditions (anxiety and depression). For the unadjusted models, current tobacco use status was used as the dependent variable with anxiety, depression, diabetes, heart disease and hypertension fitted in separate models as the independent variable. For the adjusted model, current tobacco use status was used as the dependent variable with anxiety, depression, diabetes, heart disease and hypertension as the independent variables, adjusting for the following covariates: age, sex, type of SMI, SMI duration, income tertile, education level, and country. Odds ratios with corresponding 95% confidence intervals and p-values are reported for the adjusted and unadjusted analyses. Analysis models included complete cases only; however, multiple imputations by chained equations were used to assess the robustness of the results to missing data assumptions. Ten imputed datasets were created, and the estimates from regression models carried out on each dataset were combined using Rubin’s rules. The parameter estimates from the multiple imputation (MI) models are presented in the Supplementary file.

In order to assess how the relationship between tobacco use and each of the variables anxiety, depression, diabetes, heart disease and hypertension differed between countries, a likelihood ratio test was carried out to assess whether the adjusted model with the addition of an interaction term between country and the variable of interest was a better fit than the original adjusted model. All statistical analyses were carried out using Stata version 17.0. Statistical significance was assessed at the 5% level using two-sided tests.

## RESULTS

**Figure 1:**
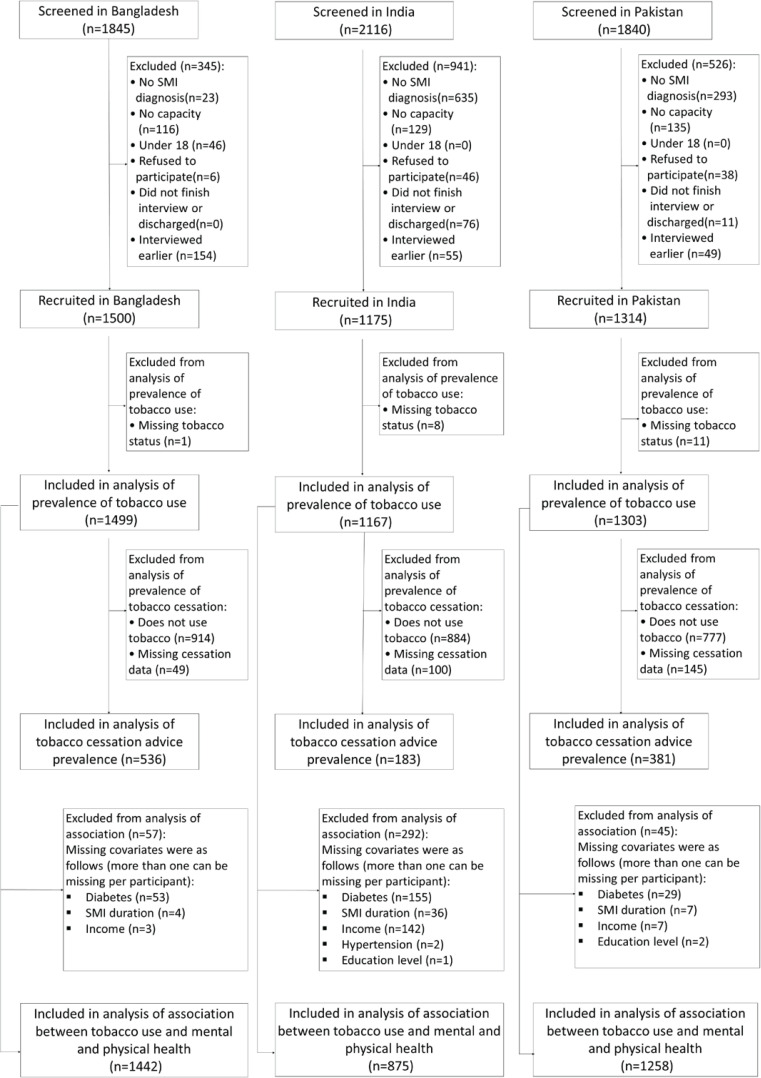
Flowchart of participants screened, recruited, and analysed for each country

Of the 5801 participants screened, 3989 (68.8%) were recruited in the survey [Bangladesh 1500 (37.6%); India 1175 (29.5%); Pakistan 1314 (32.9%)]. The number of participants included in and excluded from the survey and the various analyses (and the reasons for exclusion) are detailed in the flowchart (Figure 1).

The characteristics of study participants are presented in Table 1. Of the 3989 participants, 2359 (59.1%) were male. The average age was 35.8 years (SD=11.9; range: 18–84). Psychotic disorders were the most common type (n=1784; 44.7%) of SMI. The majority of the sample were outpatients (82.5%), as expected due to the sampling scheme, and most participants (n=1589; 41.4%) were in the lowest income tertile group.

**Table 1:** Participant characteristics summarized descriptively overall and by country

*Characteristics*	*Bangladesh (N=1500) n (%)*	*India (N=1175) n (%)*	*Pakistan (N=1314) n (%)*	*Overall (N=3989) n (%)*
**Sex**				
Male	915 (61.0)	648 (55.1)	796 (60.6)	2359 (59.1)
Female	585 (39.0)	527 (44.9)	518 (39.4)	1630 (40.9)
**Age** (years)				
Mean (SD)	31.5 (10.8)	38.8 (11.2)	38.1 (12.3)	35.8 (11.9)
Median (IQR)	30.0 (23.0–38.0)	38.0 (30.0–46.0)	36.0 (28.0–45.0)	35.0 (26.0–44.0)
Range	18.0–76.0	18.0–81.0	18.0–84.0	18.0–84.0
**Age** (years)				
18–24	434 (28.9)	123 (10.5)	159 (12.1)	716 (17.9)
25–39	732 (48.8)	538 (45.8)	603 (45.9)	1873 (47.0)
40–54	263 (17.5)	386 (32.9)	402 (30.6)	1051 (26.3)
≥55	71 (4.7)	128 (10.9)	150 (11.4)	349 (8.7)
**SMI**				
Psychotic disorder	935 (62.3)	673 (57.3)	176 (13.4)	1784 (44.7)
Major depressive disorder with psychotic features	77 (5.1)	63 (5.4)	601 (45.7)	741 (18.6)
Bipolar disorder	488 (32.5)	439 (37.4)	537 (40.9)	1464 (36.7)
**Duration of the SMI** (years)				
≤2	436 (29.1)	215 (18.3)	289 (22.0)	940 (23.6)
3–5	457 (30.5)	266 (22.6)	320 (24.4)	1043 (26.1)
6–10	332 (22.1)	299 (25.4)	299 (22.8)	930 (23.3)
>10	271 (18.1)	359 (30.6)	399 (30.4)	1029 (25.8)
Don’t know/don’t remember	4 (0.3)	36 (3.1)	7 (0.5)	47 (1.2)
**Setting**				
Inpatient	313 (20.9)	264 (22.5)	122 (9.3)	699 (17.5)
Outpatient	1187 (79.1)	911 (77.5)	1192 (90.7)	3290 (82.5)
**Education level**		N=1174	N=1312	N=3986
No formal education	151 (10.1)	141 (12.0)	257 (19.6)	549 (13.8)
Primary education	842 (56.1)	401 (34.2)	234 (17.8)	1477 (37.1)
Secondary education	234 (15.6)	228 (19.4)	273 (20.8)	735 (18.4)
Higher/more than secondary	273 (18.2)	404 (34.4)	548 (41.8)	1225 (30.7)
**Employment status** (past 12 months)		N=1174	N=1309	N=3983
Employed	439 (29.3)	522 (44.5)	507 (38.7)	1468 (36.9)
Unemployed	595 (39.7)	227 (19.3)	291 (22.2)	1113 (27.9)
Other^a^	466 (31.1)	425 (36.2)	511 (39.0)	1402 (35.2)
**Monthly household income** (US$)	N=1497	N=1032	N=1307	N=3836
Mean (SD)	224.6 (352.1)	305.2 (861.4)	198.6 (199.8)	237.4 (513.1)
Median (IQR)	176.7 (117.8–235.5)	158.9 (66.2–264.8)	148.6 (89.2–237.8)	158.9 (105.9–264.8)
Range	11.8–11777.2	0.0–16551.9	0.0–2972.7	0.0–16551.9
**Income tertile**	N=1497	N=1032	N=1307	N=3836
Low	674 (45.0)	349 (33.8)	566 (43.3)	1589 (41.4)
Middle	485 (32.4)	463 (44.9)	315 (24.1)	1263 (32.9)
High	338 (22.6)	220 (21.3)	426 (32.6)	984 (25.7)
**Marital status**				
Never married	539 (35.9)	349 (29.7)	417 (31.7)	1305 (32.7)
Currently married	818 (54.5)	711 (60.5)	747 (56.8)	2276 (57.1)
Ever married^b^	143 (9.5)	115 (9.8)	150 (11.4)	408 (10.2)

a Other includes: homemaker, student, and retired.

b Ever married includes: widowed, separated, and divorced. IQR: interquartile range.

The prevalence of current tobacco use (smoked or smokeless) for males was estimated to be 51.8% (95% CI: 48.5–55.0) in Bangladesh, 35.3% (95% CI: 31.7–39.0) in India, and 57.6% (95% CI: 54.1–61.0) in Pakistan (Table 2). Among male participants, 36.5% (n=859) reported current tobacco smoking (44.3% in Bangladesh, 25.9% in India and 36.1% in Pakistan), most (90.2%) smoking daily (Supplementary file Table A2a). Additionally, 25.2% (n=591) reported current smokeless tobacco use (16.2% in Bangladesh, 18.4% in India and 41.1% in Pakistan) (Supplementary file Table A2a). Another 45.1% of males reported past smoking.

**Table 2:** Overall and stratified tobacco use prevalence in Bangladesh, India and Pakistan

	*Bangladesh (Total=1499)*		*India (Total=1167)*		*Pakistan (Total=1303)*	
	*Females*	*Males*	*Females*	*Males*	*Females*	*Males*
**Total**	585	914	523	644	511	792
	**n/N**	**n/N**	**n/N**	**n/N**	**n/N**	**n/N**
	**% (95% CI)**	**% (95% CI)**	**% (95% CI)**	**% (95% CI)**	**% (95% CI)**	**% (95% CI)**
**Current tobacco use**	112/585	473/914	56/523	227/644	70/511	456/792
	19.2 (16.2–22.5)	51.8 (48.5–55.0)	10.7 (8.3–13.7)	35.3 (31.7–39.0)	13.7 (11.0–17.0)	57.6 (54.1–61.0)
**Ever tobacco use^a^**	115/585	537/914	62/520	293/643	84/506	514/791
	19.7 (16.6–23.1)	58.8 (55.5–61.9)	11.9 (9.4–15.0)	45.6 (41.8–49.4)	16.6 (13.6–20.1)	65.0 (61.6–68.2)
**Past tobacco use**	9/585	479/915	20/524	191/637	46/507	385/786
	1.5 (0.8–2.9)	52.4 (49.1–55.6)	3.8 (2.5–5.8)	30.0 (26.6–33.7)	9.1 (6.9–11.9)	49.0 (45.5–52.5)
**Stratified prevalence (current tobacco use)**						
**Age (years)**						
18–24	12/179	103/255	2-47	20/74	2/64	37/95
	6.7 (3.8–11.4)	40.4 (34.5–46.5)	4.3 (1.1–15.5)	27.0 (18.1–38.2)	3.1 (0.8–11.7)	38.9 (29.7–49.1)
25–39	47/263	257/469	13/216	107/320	22/199	234/397
	17.9 (13.7–23.0)	54.8 (50.3–59.3)	6.0 (3.5–10.1)	33.4 (28.5–38.8)	11.1 (7.4–16.2)	58.9 (54.0–63.7)
40–54	42/115	91/147	34/196	78/188	28/177	134/221
	36.5 (28.2–45.7)	61.9 (53.8–69.4)	17.3 (12.7–23.3)	41.5 (34.7–48.7)	15.8 (11.1–22.0)	60.6 (54.0–66.9)
≥55	11/28	22/43	7-64	22/62	18/71	51/79
	39.3 (23.3–58.0)	51.2 (36.5–65.6)	10.9 (5.3–21.2)	35.5 (24.6–48.1)	25.4 (16.6–36.7)	64.6 (53.5–74.3)
**SMI**						
Psychotic disorder	69/378	288/556	28/309	132/358	8/50	77/124
	18.3 (14.7–22.5)	51.8 (47.6–55.9)	9.1 (6.3–12.8)	36.9 (32.0–42.0)	16.0 (8.2–28.9)	62.1 (53.3–70.2)
Major depressive disorder with psychotic features	9/48	7/29	7-30	13/33	39/301	160/296
	18.8 (10.1–32.3)	24.1 (12.0–42.7)	23.3 (11.5–41.5)	39.4 (24.4–56.7)	13.0 (9.6–17.2)	54.1 (48.3–59.7)
Bipolar disorder (any)	34/159	178/329	21/184	82/253	23/160	219/372
	21.4 (15.7–28.4)	54.1 (48.7–59.4)	11.4 (7.6–16.9)	32.4 (26.9–38.4)	14.4 (9.7–20.7)	58.9 (53.8–63.8)
**Duration of the SMI** (years)						
≤2	34/204	113/231	4/102	41/112	14/137	70/151
	16.7 (12.2–22.4)	48.9 (42.5–55.3)	3.9 (1.5–10.0)	36.6 (28.2–45.9)	10.2 (6.1–16.5)	46.4 (38.6–54.3)
3–5	36/164	142/293	17/119	47/146	9/111	116/208
	22.0 (16.3–28.9)	48.5 (42.8–54.2)	14.3 (9.1–21.8)	32.2 (25.1–40.2)	8.1 (4.3–14.9)	55.8 (49.0–62.4)
6–10	26/124	115/208	19/131	56/164	18/105	115/189
	21.0 (14.7–29.0)	55.3 (48.5–61.9)	14.5 (9.4–21.6)	34.1 (27.3–41.7)	17.1 (11.1–25.6)	60.8 (53.7–67.5)
>10	16/92	102/179	12/150	78/207	28/155	154/240
	17.4 (10.9–26.5)	57.0 (49.6–64.0)	8.0 (4.6–13.6)	37.7 (31.3–44.5)	18.1 (12.8–24.9)	64.2 (57.9–70.0)
**Income tertile**						
Low	55/254	212/419	22/157	86/191	29/203	214/358
	21.7 (17.0–27.1)	50.6 (45.8–55.4)	14.0 (9.4–20.4)	45.0 (38.1–52.1)	14.3 (10.1–19.8)	59.8 (54.6–64.7)
Middle	33/192	159/293	21/205	84/255	16/137	99/176
	17.2 (12.5–23.2)	54.3 (48.5–59.9)	10.2 (6.8–15.2)	32.9 (27.4–38.9)	11.7 (7.3–18.2)	56.3 (48.8–63.4)
High	23/137	101/201	4-77	45/140	25/167	142/255
	16.8 (11.4–24.0)	50.2 (43.4–57.1)	5.2 (2.0–13.0)	32.1 (24.9–40.3)	15.0 (10.3–21.2)	55.7 (49.5–61.7)

a Ever tobacco use includes those who currently use tobacco or have smoked tobacco in the past.

The prevalence of current tobacco use for females was lower than for males; 19.2% (95% CI: 16.2–22.5) in Bangladesh, 10.7% (95% CI: 8.3–13.7) in India, and 13.7% (95% CI: 11.0–17.0) in Pakistan (Table 2). Among female participants, 2.5% (n=40) reported current tobacco smoking (0.5% in Bangladesh, 1.5% in India and 5.7% in Pakistan), most (84.6%) smoking daily (Supplementary file Table A2b). Additionally, 13.2% (n=213) reported current smokeless tobacco use (19.1% in Bangladesh, 9.9% in India and 9.6% in Pakistan) (Supplementary file Table A2b). Another 4.6% of female participants reported past smoking. Tobacco use was most prevalent amongst the age groups of 40–54 and ≥55 years, and least prevalent among participants aged 18–24 years (Table 2). Further information on tobacco use is presented in Supplementary file Table A1.

Approximately 38.4% (n=422) of tobacco users (40.7%, 49.2%, and 29.9% in Bangladesh, India and Pakistan, respectively) reported receiving advice to quit during a healthcare visit in the past 12 months (Table 3). In Bangladesh, the prevalence of receiving advice to quit tobacco was much lower for those aged ≥55 years, than the other age groups. In contrast, in India, those aged ≥55 years had the highest prevalence of receiving advice to quit (Supplementary file Table A3b). In all three countries, the prevalence of receiving advice to quit was higher for males than females (Supplementary file Tables A3a and A3b).

**Table 3:** Participant tobacco cessation advice summarized descriptively overall and by country

	*Bangladesh n (%)*	*India n (%)*	*Pakistan n (%)*	*Overall n (%)*
**Currently use tobacco** (smoked or smokeless)	**N=585**	**N=283**	**N=526**	**N=1394**
**Advised to quit using tobacco in the past 12 months**	**N=536**	**N=183**	**N=381**	**N=1100**
Yes	218 (40.7)	90 (49.2)	114 (29.9)	422 (38.4)
No	318 (59.3)	93 (50.8)	267 (70.1)	678 (61.6)
**Currently smoke tobacco**	**N=408**	**N=176**	**N=315**	**N=899**
**Smokers who tried to quit in past 12 months**	**N=408**	**N=141**	**N=290**	**N=839**
Yes	63 (15.4)	73 (51.8)	108 (37.2)	244 (29.1)
No	345 (84.6)	68 (48.2)	182 (62.8)	595 (70.9)
**Smokers who received cessation advice in the past 12 months**	**N=408**	**N=161**	**N=306**	**N=875**
Yes	198 (48.5)	68 (42.2)	60 (19.6)	326 (37.3)
No	206 (50.5)	71 (44.1)	180 (58.8)	457 (52.2)
No healthcare visit in past 12 months	4 (1.0)	22 (13.7)	66 (21.6)	92 (10.5)

Among smokers, 29.1% (n=244) made at least one quit attempt in the past 12 months; this differed by country with just over half (51.8%) making a quit attempt in India, compared with only 15.4% in Bangladesh, and 37.2% in Pakistan (Table 3).

In India, participants who use tobacco scored higher on the PHQ-9, and a larger proportion of tobacco users (n=108; 38.2%) were classified as having moderate or severe depression compared to those who do not use tobacco (19.9%) (Table 4). Similarly, a larger proportion of tobacco users were classified as having moderate or severe anxiety according to the GAD-7 score; 30.0% (n=85) of tobacco users versus 13.9% (n=123) of those not using tobacco. These differences were not observed in other countries. Descriptive summaries of candidate variables presented by country and whether or not the participant currently smokes tobacco are presented in Supplementary file Table A4.

**Table 4:** Descriptive summaries of candidate variables presented by country and whether or not the participant currently uses tobacco

	Bangladesh (N=1499)*		India (N=1167)*		Pakistan (N=1303)*		*Overall (N=3969)*	
	*Use tobacco n (%)*	*Do not use tobacco n (%)*	*Use tobacco n (%)*	*Do not use tobacco n (%)*	*Use tobacco n (%)*	*Do not use tobacco n (%)*	*Use tobacco n (%)*	*Do not use tobacco n (%)*
**Total**	**585**	**914**	**283**	**884**	**526**	**777**	**1394**	**2575**
**Depression** (PHQ-9 score)								
Mean (SD)	10.5 (4.4)	10.8 (4.8)	8.2 (7.7)	5.1 (6.2)	12.5 (6.8)	13.1 (6.9)	10.8 (6.3)	9.5 (6.9)
Median (IQR)	10.0 (8.0–13.0)	10.0 (8.0–14.0)	7.0 (1.0–15.0)	2.0 (0.0–8.0)	12.0 (7.0–18.0)	13.0 (8.0–18.0)	10.0 (6.0–15.0)	9.0 (4.0–14.0)
Range	0.0–24.0	0.0–27.0	0.0–27.0	0.0–27.0	0.0–27.0	0.0–27.0	0.0–27.0	0.0–27.0
**PHQ-9 group**								
No/mild depression	238 (40.7)	370 (40.5)	175 (61.8)	708 (80.1)	195 (37.1)	248 (31.9)	608 (43.6)	1326 (51.5)
Moderate/severe depression	347 (59.3)	544 (59.5)	108 (38.2)	176 (19.9)	331 (62.9)	529 (68.1)	786 (56.4)	1249 (48.5)
**Anxiety** (GAD-7 score)								
Mean (SD)	8.1 (3.8)	8.0 (3.9)	6.5 (6.2)	3.9 (5.0)	9.7 (5.0)	10.1 (5.2)	8.4 (5.0)	7.2 (5.3)
Median (IQR)	8.0 (6.0–11.0)	8.0 (6.0–10.0)	5.0 (1.0–12.0)	2.0 (0.0–6.0)	9.0 (6.0–13.0)	10.0 (7.0–14.0)	8.0 (5.0–12.0)	7.0 (3.0–11.0)
Range	0.0–21.0	0.0–21.0	0.0–21.0	0.0–21.0	0.0–21.0	0.0–21.0	0.0–21.0	0.0–21.0
**GAD-7 group**								
No/mild anxiety	366 (62.6)	611 (66.8)	198 (70.0)	761 (86.1)	270 (51.3)	355 (45.7)	834 (59.8)	1727 (67.1)
Moderate/severe anxiety	219 (37.4)	303 (33.2)	85 (30.0)	123 (13.9)	256 (48.7)	422 (54.3)	560 (40.2)	848 (32.9)
**Diabetes**	**N=568**	**N=878**	**N=242**	**N=770**	**N=514**	**N=760**	**N=1324**	**N=2408**
Yes	47 (8.3)	80 (9.1)	49 (20.2)	115 (14.9)	47 (9.1)	73 (9.6)	143 (10.8)	268 (11.1)
No	521 (91.7)	798 (90.9)	193 (79.8)	655 (85.1)	467 (90.9)	687 (90.4)	1181 (89.2)	2140 (88.9)
**Hypertension**				**N=882**				**N=2573**
Yes	65 (11.1)	78 (8.5)	38 (13.4)	101 (11.5)	130 (24.7)	199 (25.6)	233 (16.7)	378 (14.7)
No	520 (88.9)	836 (91.5)	245 (86.6)	781 (88.5)	396 (75.3)	578 (74.4)	1161 (83.3)	2195 (85.3)
**Heart disease**								
Yes	7 (1.2)	13 (1.4)	6 (2.1)	27 (3.1)	38 (7.2)	35 (4.5)	51 (3.7)	75 (2.9)
No	578 (98.8)	901 (98.6)	277 (97.9)	857 (96.9)	488 (92.8)	742 (95.5)	1343 (96.3)	2500 (97.1)

* Tobacco use status was missing for n=20 participants (Bangladesh, n=1; India, n=8; Pakistan, n=11).

**Figure 2:**
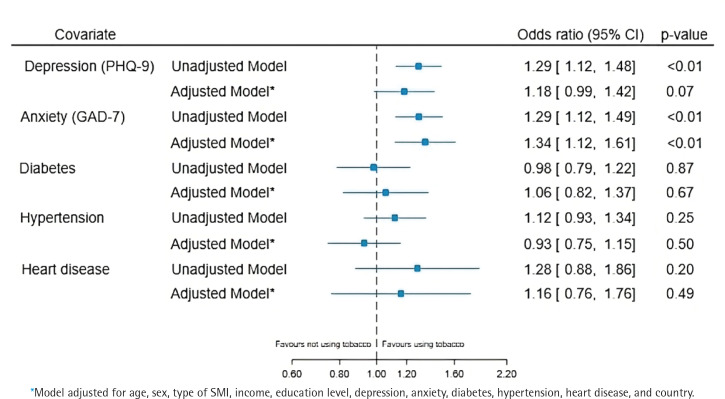
Association of tobacco use with physical and mental health conditions

Figure 2 presents the results of the unadjusted and adjusted analyses assessing the association between tobacco use and the variables of interest. There was weak evidence to suggest an association between tobacco use and depression, with an adjusted odds ratio (AOR) of 1.18 (95% CI: 0.99–1.42; p=0.07), whereas there was strong evidence of an association between tobacco use and anxiety (AOR=1.34; 95% CI: 1.12–1.61; p<0.01). There was no evidence of an association between tobacco use and the remaining variables of interest.

There was strong evidence that the association between tobacco use and the severity of depression (p<0.01) and anxiety (p<0.01) varied among countries. There was no evidence that between countries there was variation in the association between tobacco use and diabetes (p=0.23), hypertension (p=0.83), and heart disease (p=0.15). The analysis using multiple imputation by chained equations did not change the interpretation of the results (Supplementary file Table A5).

## DISCUSSION

Our study found that people with SMI who live in South Asia are more likely to consume tobacco than the general population. Among men, nearly half (46.8%) and among women, one-sixth (15%) were consuming tobacco on a regular basis at the time of our survey. The distribution of tobacco use across different age groups and genders across different types of tobacco products was similar to what has been observed in the general population in these countries. On the other hand, just over a third of these tobacco users (38.4%) received advice to quit tobacco in the previous 12 months, despite being in contact with healthcare professionals. Hence, less than a third of smokers made any attempt to stop smoking in the past 12 months. We also found that tobacco use in this population was strongly associated with the severity of anxiety symptoms.

Our estimated prevalence of tobacco use among people with SMI is consistent with previously conducted systematic reviews and meta-analyses^20,21^. In a meta-analysis of 19 studies including 7527 people with SMI, the prevalence of tobacco use disorder/nicotine dependence was 65% in people with schizophrenia, 46.3% in those with bipolar disorder, and 33.4% in those with major depressive disorder^20^. Another meta-analysis of 21 studies found that those at clinically high risk of psychosis are more likely to smoke than healthy controls (OR=2.22; 95% CI: 1.74–2.84, p<0.01)^21^. Out of 40 studies in these two metanalyses, only 3 included research conducted in LMICs.

Country-specific variations in the prevalence of smoking tobacco were also found within the countries examined during this study, aligning with existing literature. For instance, the reported prevalence of smoked tobacco varied from 27% in a study in people with SMI in the UK^22^, 40% in a study in Sri Lanka^23^, 48% in the US^24^, and 55% in France^25^. These differences in smoking prevalence between countries may be related to social influences and contextual differences, such as cultural norms and attitudes towards tobacco use^26^. Additionally, national policies pertaining to tobacco, encompassing aspects such as advertising, taxation, and tobacco sales availability, can influence smoking rates. Furthermore, the health literacy levels of populations within each country may also contribute to the differences in smoking prevalence^27,28^. Limited access to healthcare and insufficient guidance on health behavior modifications can impact tobacco use rates as well. Moreover, the presence of stigma surrounding mental health may further affect smoking prevalence, particularly among those with SMI^29^.

Supporting smoking cessation in people with severe mental illness requires a comprehensive approach that considers both the mental health and tobacco dependence aspects. Integrated interventions, such as mental health services that include smoking cessation support, along with public health campaigns and policy changes, can play a crucial role in reducing smoking rates and improving the overall health and well-being of this vulnerable population.

The lower prevalence of tobacco use among the younger age group might be associated with generational trends, social norms and attitudes toward smoking^30^. The higher prevalence of tobacco use in older age groups could be attributed to historical patterns of smoking prevalence. In the past, smoking was more socially acceptable, and tobacco was heavily marketed, leading to higher smoking rates among older generations. On the other hand, younger individuals may have grown up in an era with increased awareness of the health risks associated with smoking, leading to lower tobacco use rates among them. Recognizing the lower prevalence of tobacco use among those aged 18–24 years presents an opportunity for public health initiatives to build upon this positive trend by implementing targeted anti-smoking campaigns, and education about the dangers of smoking^31^.

We found an unmet need for tobacco cessation advice across all countries. India had a higher proportion of individuals who have attempted to quit tobacco, which may be associated with the country’s higher rate of providing advice for quitting. While this correlation does not establish a causal relationship, it suggests that offering guidance and support to those seeking to quit tobacco use may have a positive impact on encouraging quit attempts^32^. These results highlight the significance of implementing comprehensive tobacco cessation programs, focusing on providing accessible and tailored support to individuals who wish to quit.

### Implications

Across most countries in the world, including Bangladesh, India and Pakistan, smoking rates are declining in the general population primarily as a result of coordinated international efforts of the last two decades^13^. However, a corresponding decline has not been seen in people with mental illnesses^33^. Our findings confirm this lack of progress for people with SMI in Bangladesh, India and Pakistan. This is of particular concern, as people with SMI are likely to die 10–15 years before the life expectancy in their respective country and tobacco use is most likely to be responsible for this disparity. In addition to the increased cardiovascular, respiratory and cancer-related mortality, tobacco use is also responsible for the recurrence of mental health conditions^34^. This disproportionate impact of tobacco on the mental and physical health of people with mental illness makes the identification and treatment of tobacco addiction a necessary practice which should be integrated into regular mental healthcare^35^. The need for offering tobacco cessation and promoting smoke-free environments within mental health institutions should be included in the country’s mental health policies. Furthermore, media campaigns should highlight the need to address this disparity and dispel some of the myths about treating tobacco addiction in people with mental illness.

### Strengths and limitations

This study has a number of strengths. This is the first large-scale multi-country survey ever conducted in South Asia reporting tobacco use and its association with mental and physical health in people with SMI. To assess tobacco use, we used questions employed in STEPS population surveys in these countries, which allows for direct comparisons. Similarly, standardized tools for anxiety and depression were used to assess the severity of mental illness.

There are also several limitations. The survey was conducted in a purposive and limited sample of mental health institutions and therefore the prevalence of tobacco use cannot be generalized to the rest of the three countries. However, people with SMI in Bangladesh, India and Pakistan are highly likely to be treated at some point in the course of their illness in institutions similar to the ones selected in this survey. In the absence of any mental health services in primary and secondary care in South Asia, most people with SMI are seen directly at specialized mental health institutions. Hence, the survey participants are likely to be similar to people with SMI in those countries. Some of the survey participants were interviewed at the peak of COVID-19 pandemic, particularly those based in India, which might have influenced the findings. The pandemic influenced people’s tobacco use behaviors with some taking up/increasing its use while others quitting/decreasing its use^36^. However, the pandemic influence was bidirectional and it might not have shifted the prevalence in any one direction. Furthermore, the pandemic would not have influenced the proportion of people receiving quit advice if visiting a health professional. We assessed tobacco use behavior subjectively, which raises questions about the validity of our findings. However, almost all Global Tobacco Surveillance Systems assess tobacco use through such questionnaires and this approach is considered reliable. The observational nature of the study means that causal inferences could not be made from the analysis of associations between tobacco use and the variables of interest.

## CONCLUSIONS

People with SMI have a higher prevalence of tobacco use compared to the general population. The severity of their mental health illness may be associated with their tobacco use. On the other hand, the high level of tobacco use in people with SMI is likely to be responsible for the high proportion of cardiovascular- and cancer-related deaths in this population. Our findings highlight the importance of offering cessation advice for people with SMI and in general supporting smoke-free environments in mental health institutions.

## ^+^IMPACT research team

### Program Management Group

Department of Mental Health and Substance; World Health Organization, Switzerland: Neerja Chowdhary University of York, United Kingdom: Rachel Churchill, Razia Fatima, Simon Gilbody, Cate Cowton, Rowena Jacobs, Najma Siddiqi, Catherine Hewitt, Dave Ekers ARK Foundation, Bangladesh: Rumana Huque London School of Economics and Political Science, United Kingdom: David McDaid University of Leeds, United Kingdom: Tolib Mirzoev National Institute of Mental Health and Neurosciences (NIMHANS), India: Pratima Murthy International Centre for Diarrhoeal Disease Research, Bangladesh (ICDDR,B): Aliya Naheed Rawalpindi Medical University, Pakistan: Asad Nizami The University of Dundee, United Kingdom: Jan Boehnke University of Southampton: Richard Holt

### Research Fellows and Research Associates

National Institute of Mental Health and Neurosciences (NIMHANS), India: Arun Kandasamy, Faiza Aslam, Krishna Prasad, Naseer Bhat, Nithyananda S. ARK Foundation, Bangladesh: Asiful Haidar Chowdhury King’s College London, United Kingdom: Brendon Stubbs University College London: David Shiers University of York, United Kingdom: Gerardo Zavala, Kamran Siddiqi, Nicky Traynor, Noortje Uphoff, Noreen Mdege, Papiya Mazumdar Institute of Psychiatry, Pakistan: Humaira Khalid, Nida Afsheen University of Leeds, United Kingdom: Judy Wright International Centre for Diarrhoeal Disease Research, Bangladesh (ICDDR,B): Nabila Ashraf, Nantu Chakma University of Sussex, United Kingdom: Papreen Nahar

### Research Assistants

Institute of Psychiatry, Pakistan: Rubab Ayesha, Najma Hayat, Zaheen Amin, Ayaz Ali, Fakiha Shabbir, Kousar Ishaq, Zauraiz Lone, Bilal Ahmed Khan, Humaira Bibi.

## Data Availability

The data supporting this research are available from the authors on reasonable request.

## References

[1] Heller T, Roccoforte JA, Hsieh K, Cook JA, Pickett SA (1997). Benefits of support groups for families of adults with severe mental illness. Am J Orthopsychiatry.

[2] Walker ER, McGee RE, Druss BG (2015). Mortality in mental disorders and global disease burden implications: a systematic review and meta-analysis. JAMA Psychiatry.

[3] Lawrence D, Kisely S, Pais J (2010). The epidemiology of excess mortality in people with mental illness. Can J Psychiatry.

[4] Scott KM, Von Korff M, Alonso J (2009). Mental–physical co-morbidity and its relationship with disability: results from the World Mental Health Surveys. Psychol Med.

[5] Osborn DPJ, Nazareth I, King MB (2007). Physical activity, dietary habits and Coronary Heart Disease risk factor knowledge amongst people with severe mental illness: a cross sectional comparative study in primary care. Soc Psychiat Epidemiol.

[6] Afzal M, Siddiqi N, Ahmad B (2021). Prevalence of overweight and obesity in people with severe mental illness: systematic review and meta-analysis. Front Endocrinol (Lausanne).

[7] de Leon J, Diaz FJ (2005). A meta-analysis of worldwide studies demonstrates an association between schizophrenia and tobacco smoking behaviors. Schizophr Res.

[8] Cook BL, Wayne GF, Kafali EN, Liu Z, Shu C, Flores M (2014). Trends in smoking among adults with mental illness and association between mental health treatment and smoking cessation. JAMA.

[9] Lawn S, Pols R (2005). Smoking bans in psychiatric inpatient settings? A review of the research. Aust N Z J Psychiatry.

[10] Thornton LK, Baker AL, Lewin TJ (2012). Reasons for substance use among people with mental disorders. Addict Behav.

[11] Szatkowski L, McNeill A (2015). Diverging trends in smoking behaviors according to mental health status. Nicotine Tob Res.

[12] Johnson JL, Ratner PA, Malchy LA (2010). Gender-specific profiles of tobacco use among non-institutionalized people with serious mental illness. BMC Psychiatry.

[13] World Health Organization WHO Report on the Global Tobacco Epidemic, 2021: Addressing new and emerging products.

[14] Zavala GA, Todowede O, Mazumdar P (2022). Effectiveness of interventions to address obesity and health risk behaviours among people with severe mental illness in low- and middle-income countries (LMICs): a systematic review and meta analysis. Global Mental Health.

[15] Mazumdar P, Zavala G, Aslam F (2023). IMPACT smoking cessation support for people with severe mental illness in South Asia (IMPACT 4S): A protocol for a randomised controlled feasibility trial of a combined behavioural and pharmacological support intervention. PLoS One.

[16] Badekar A, Rajalu BM, Muliyala KP, Sharma P, Chand PK, Murthy P (2022). Prevalence and severity of tobacco use and access to tobacco cessation among tertiary care psychiatric in-patients in India. Indian J Psychiatry.

[17] Chandra PS, Carey MP, Carey KB, Jairam KR, Girish NS, Rudresh HP (2005). Prevalence and correlates of tobacco use and nicotine dependence among psychiatric patients in India. Addict Behav.

[18] Zavala GA, Prasad-Muliyala K, Aslam F (2020). Prevalence of physical health conditions and health risk behaviours in people with severe mental illness in South Asia: protocol for a cross-sectional study (IMPACT SMI survey). BMJ Open.

[19] Zavala GA, Haidar-Chowdhury A, Prasad-Muliyala K (2023). Prevalence of physical health conditions and health risk behaviours in people with severe mental illness in South Asia: multi-country cross-sectional survey. BJPsych Open.

[20] Fornaro M, Carvalho AF, De Prisco M (2022). The prevalence, odds, predictors, and management of tobacco use disorder or nicotine dependence among people with severe mental illness: systematic review and meta-analysis. Neurosci Biobehav Rev.

[21] De Micheli A, Provenzani U, Solmi M (2023). Prevalence of tobacco smoking in people at clinical high-risk for psychosis: systematic review and meta-analysis. Schizophr Res.

[22] Peckham E, Allgar V, Crosland S (2021). Investigating smoking and nicotine dependence among people with severe mental illness during the COVID-19 pandemic: analysis of linked data from a UK Closing the Gap cohort. BJPsych Open.

[23] Edrisinghe N, Wijesinghe CA, Williams SS, Kuruppuarachchi KALA (2014). Tobacco smoking in persons with schizophrenia followed up at a teaching hospital in Sri Lanka. Sri Lanka Journal of Psychiatry.

[24] Kalkhoran S, Thorndike AN, Rigotti NA, Fung V, Baggett TP (2019). Cigarette smoking and quitting-related factors among US adult health center patients with serious mental illness. J Gen Intern Med.

[25] Denis F, Milleret G, Wallenhorst T, Carpentier M, Rude N, Trojak B (2019). Oral health in schizophrenia patients: a French multicenter cross-sectional study. Presse Med.

[26] Polanska K, Znyk M, Kaleta D (2022). Susceptibility to tobacco use and associated factors among youth in five central and eastern European countries. BMC Public Health.

[27] Siddiqi K, Siddiqui F, Boeckmann M (2022). Attitudes of smokers towards tobacco control policies: findings from the Studying Tobacco users of Pakistan (STOP) survey. Tob Control.

[28] Mishu MP, Siddiqui F, Shukla R, Kanaan M, Dogar O, Siddiqi K (2021). Predictors of cigarette smoking, smokeless tobacco consumption, and use of both forms in adolescents in South Asia: a secondary analysis of the Global Youth Tobacco Surveys. Nicotine Tob Res.

[29] Cocks N, Brophy L, Segan C, Stratford A, Jones S, Castle D (2019). Psychosocial factors affecting smoking cessation among people living with schizophrenia: a lived experience lens. Front Psychiatry.

[30] World Health Organization (2019). WHO global report on trends in prevalence of tobacco use 2000-2025.

[31] Flor LS, Reitsma MB, Gupta V, Ng M, Gakidou E (2021). The effects of tobacco control policies on global smoking prevalence. Nat Med.

[32] Davila EP, Zhao W, Byrne M (2009). Correlates of smoking quit attempts: Florida Tobacco Callback Survey, 2007. Tob Induc Dis.

[33] Berg SA, Sentir AM, Cooley BS, Engleman EA, Chambers RA (2014). Nicotine is more addictive, not more cognitively therapeutic in a neurodevelopmental model of schizophrenia produced by neonatal ventral hippocampal lesions. Addict Biol.

[34] Scott JG, Matuschka L, Niemelä S, Miettunen J, Emmerson B, Mustonen A (2018). Evidence of a causal relationship between smoking tobacco and schizophrenia spectrum disorders. Front Psychiatry.

[35] Mendelsohn CP, Kirby DP, Castle DJ (2015). Smoking and mental illness. An update for psychiatrists. Australas Psychiatry.

[36] Siddiqi K, Siddiqui F, Khan A (2021). The impact of COVID-19 on smoking patterns in Pakistan: findings from a longitudinal survey of smokers. Nicotine Tob Res.

