# Supplementary material for: Tobacco use in people with severe mental illness: Findings from a multi-country survey of mental health institutions in South Asia
Source: Tob Induc Dis. 2023 Dec 14;21:166. doi: 10.18332/tid/174361 (PMC10720264; doi:10.18332/tid/174361)
Supplement: Supplementary file 1 [file TID-21-166-s1.pdf]

Appendix

A1 Tobacco use – All Participants

In total, 22.6% (n=899) of participants reported that they currently smoke tobacco products (27.2% in Bangladesh, 15.0% in India and 24.2% in Pakistan). Of those who smoke, 90.0% (n=807) reported that they smoke daily (96.6% in Bangladesh, 77.0% in India and 88.6% in Pakistan). 28.6% (n=1130) of participants reported that they have smoked tobacco in the past. Additionally, 20.3% (n=804) of participants reported that they currently use smokeless tobacco products (17.3% in Bangladesh, 14.6% in India and 28.7% in Pakistan) (Table A1).

Table A1: Participant tobacco use summarised descriptively overall and by country.

	**Bangladesh (n=1500)**	**India (n=1175)**	**Pakistan (n=1314)**	**Overall (n=3989)**
**Currently smokes tobacco products, n (%)**				
Number with data	*1500 (100)*	*1175 (100)*	*1304 (99.2)*	*3979 (99.7)*
Yes	408 (27.2)	176 (15.0)	315 (24.2)	899 (22.6)
No	1092 (72.8)	999 (85.0)	989 (75.8)	3080 (77.4)
**Currently smokes tobacco products daily, n (% of those who smoke)**				
Number with data	*408 (100)*	*174 (98.8)*	*315 (100)*	*897 (99.8)*
Yes	394 (96.6)	134 (77.0)	279 (88.6)	807 (90.0)
No	14 (3.4)	40 (23.0)	36 (11.4)	90 (10.0)
**Age when first started smoking**				
Number with data (% of those who smoke)	*408 (100)*	*156 (88.6)*	*314 (99.7)*	*878 (97.7)*
Mean (SD)	16.9 (3.4)	19.2 (7.7)	20.6 (7.1)	18.6 (6.1)
Median (IQR)	16.0 (15.0-18.0)	18.0 (15.0-22.0)	20.0 (16.0-24.0)	18.0 (15.0-20.0)
Min, Max	8.0, 37.0	1.0, 50.8	4.0, 52.0	1.0, 52.0
**Average number smoked per day:**				
**Manufactured cigarettes**				
Number with data (% of smokers)	*385 (94.4)*	*83 (47.2)*	*278 (88.3)*	*746 (83.0)*
Mean (SD)	8.2 (6.1)	8.8 (13.4)	12.5 (12.6)	9.8 (10.1)
Median (IQR)	7.0 (5.0-10.0)	4.3 (1.4-10.0)	10.0 (3.0-20.0)	7.0 (4.0-12.0)
Min, Max	0.3, 70.0	0.1, 77.0	0.1, 80.0	0.1, 80.0
**Hand-rolled cigarettes**	*25 (6.1)*	*53 (30.1)*	*26 (8.3)*	*104 (11.6)*

Number with data (% of smokers)	15.8 (11.7)	15.9 (17.3)	5.9 (6.9)	13.4 (14.6)
Mean (SD)	15.0 (5.0-25.0)	12.0 (6.0-20.0)	3.0 (1.0-10.0)	10.0 (3.5-20.0)
Median (IQR)			0.1, 20.0	
Min, Max	2.0, 50.0	0.3, 100.0		0.1, 100.0
**Cigars, cheroots, cigarillos**				
Number with data (% of smokers)	*0 (0)*	*0 (0)*	*28 (8.8)*	*28 (3.1)*
Mean (SD)	-	-	3.8 (4.4)	3.8 (4.4)
Median (IQR)	-	-	3.0 (1.0-4.0)	3.0 (1.0-4.0)
Min, Max	-	-	0.1, 20.0	0.1, 20.0
**Number of Shisha sessions**				
Number with data (% of smokers)	*0 (0)*	*0 (0)*	*3 (1.0)*	*3 (0.3)*
Mean (SD)	-	-	0.2 (0.1)	0.2 (0.1)
Median (IQR)	-	-	0.1 (0.1-0.3)	0.1 (0.1-0.3)
Min, Max	-	-	0.1, 0.3	0.1, 0.3
**Previously smoked tobacco products, n (%)**				
Number with data	*1500 (100)*	*1161 (98.8)*	*1293 (98.4)*	*3954 (99.1)*
Yes	488 (32.5)	211 (18.2)	431 (33.3)	1130 (28.6)
No	1012 (67.5)	950 (81.8)	862 (66.7)	2824 (71.4)
**Previously smoked tobacco products daily, n (% of those who previously smoked)**				
Number with data	*485 (99.4)*	*208 (98.6)*	*431 (100)*	*1124 (99.5)*
Yes	461 (95.1)	156 (75.0)	353 (81.9)	970 (86.3)
No	24 (4.9)	52 (25.0)	78 (18.1)	154 (13.7)
**Currently uses smokeless tobacco products, n (%)**				
Number with data	*1499 (99.9)*	*1164 (99.1)*	*1302 (99.1)*	*3965 (99.4)*
Yes	260 (17.3)	170 (14.6)	374 (28.7)	804 (20.3)
No	1239 (82.7)	994 (85.4)	928 (71.3)	3161 (79.7)
**Currently uses smokeless tobacco products daily, n (% of those currently using smokeless tobacco)**	*259 (99.6)*	*169 (99.4)*	*374 (100)*	*802 (99.8)*
Number with data	220 (84.9)	120 (71.0)	337 (90.1)	677 (84.4)
Yes	39 (15.1)	49 (29.0)	37 (9.9)	125 (15.6)

No				

A2 Smoking – All Participants

Table A2a: Participant tobacco use summarised descriptively overall and by country for **male participants** only.

	**Bangladesh (n=915)**	**India (n=648)**	**Pakistan (n=796)**	**Overall (n=2359)**
**Currently smokes tobacco products, n (%)**				
Number with data	*915 (100)*	*648 (100)*	*792 (99.5)*	*2355 (99.8)*
Yes	405 (44.3)	168 (25.9)	286 (36.1)	859 (36.5)
No	510 (55.7)	480 (74.1)	506 (63.9)	1496 (63.5)
**Currently smokes tobacco products daily, n (% of those who smoke)**				
Number with data	*405 (100)*	*167 (99.4)*	*286 (100)*	*858 (99.9)*
Yes	392 (96.8)	130 (77.8)	252 (88.1)	774 (90.2)
No	13 (3.2)	37 (22.2)	34 (11.9)	84 (9.8)
**Age when first started smoking**				
Number with data (% of those who smoke)	*405 (100)*	*149 (88.7)*	*285 (99.7)*	*839 (97.7)*
Mean (SD)	16.9 (3.4)	19.2 (7.9)	20.2 (6.4)	18.4 (5.7)
Median (IQR)	16.0 (15.0-18.0)	18.0 (15.0-22.0)	20.0 (16.0-23.0)	18.0 (15.0-20.0)
Min, Max	8.0, 37.0	1.0, 50.8	4.0, 50.0	1.0, 50.8
**Average number smoked per day:**				
**Manufactured cigarettes**				
Number with data and smoked non-zero value (% of smokers)	*382 (94.3)*	*80 (47.6)*	*266 (93.0)*	*728 (84.7)*
Mean (SD)	8.2 (6.1)	8.5 (13.4)	12.6 (12.3)	9.8 (10.0)
Median (IQR)	7.0 (5.0-10.0)	4.1 (1.7-10.0)	10.0 (3.0-20.0)	7.0 (4.0-12.0)
Min, Max	0.3, 70.0	0.1, 77.0	0.1, 80.0	0.1, 80.0
**Hand-rolled cigarettes**	*25 (6.2)*	*52 (31.0)*	*25 (8.7)*	*102 (11.9)*

Number with data and smoked non-				
zero value (% of smokers)	15.8 (11.7)	16.0 (17.4)	5.3 (6.4)	13.3 (14.7)
Mean (SD)	15.0 (5.0-25.0)	12.0 (5.5-20.0)	3.0 (1.0-10.0)	10.0 (3.0-20.0)
Median (IQR)	2.0, 50.0	0.3, 100.0	0.1, 20.0	0.1, 100.0
Min, Max				
**Cigars, cheroots, cigarillos**				
Number with data and smoked non-	*0 (0)*	*0 (0)*	*12 (4.2)*	*12 (1.4)*
zero value (% of smokers)				
Mean (SD)	-	-	3.7 (5.9)	3.7 (5.9)
Median (IQR)	-	-	1.0 (0.1-4.0)	1.0 (0.1-4.0)
Min, Max	-	-	0.1, 20.0	0.1, 20.0
**Number of Shisha sessions**				
Number with data and smoked non-	*0 (0)*	*0 (0)*	*3 (1.0)*	*3 (0.3)*
zero value (% of smokers)				
Mean (SD)	-	-	0.2 (0.1)	0.2 (0.1)
Median (IQR)	-	-	0.1 (0.1-0.3)	0.1 (0.1-0.3)
Min, Max	-	-	0.1, 0.3	0.1, 0.3
**Previously smoked tobacco products, n (%)**				
Number with data	*915 (100)*	*637 (98.3)*	*786 (98.7)*	*2338 (99.1)*
Yes	479 (52.3)	191 (30.0)	385 (49.0)	1055 (45.1)
No	436 (47.7)	446 (70.0)	401 (51.0)	1283 (54.9)
**Previously smoked tobacco products daily, n (% of those who previously smoked)**				
Number with data	*476 (52.0)*	*189 (99.0)*	*385 (100)*	*1050 (99.5)*
Yes	457 (96.0)	145 (76.7)	322 (83.6)	924 (88.0)
No	19 (4.0)	44 (23.3)	63 (16.4)	126 (12.0)
**Currently uses smokeless tobacco products, n (%)**	*914 (99.9)*	*641 (98.9)*	*791 (99.4)*	*2346 (99.4)*
Number with data	148 (16.2)	118 (18.4)	325 (41.1)	591 (25.2)
Yes	766 (83.8)	523 (81.6)	466 (58.9)	1755 (74.8)

No				
**Currently uses smokeless tobacco products daily, n (% of those currently using smokeless tobacco)**				
Number with data	*148 (100)*	*117 (99.2)*	*325 (100)*	*590 (99.8)*
Yes	117 (79.1)	85 (72.6)	293 (90.2)	495 (83.9)
No	31 (20.9)	32 (27.4)	32 (9.8)	95 (16.1)

Table A2b: Participant tobacco use summarised descriptively overall and by the country for **female participants** only.

	**Bangladesh (n=585)**	**India (n=527)**	**Pakistan (n=518)**	**Overall (n=1630)**
**Currently smokes tobacco products, n (%)**				
Number with data	*585 (100)*	*527 (100)*	*512 (98.8)*	*1624 (99.6)*
Yes	3 (0.5)	8 (1.5)	29 (5.7)	40 (2.5)
No	582 (99.5)	519 (98.5)	483 (94.3)	1584 (97.5)
**Currently smokes tobacco products daily, n (% of those who smoke)**				
Number with data	*3 (100)*	*7 (87.5)*	*29 (100)*	*39 (97.5)*
Yes	2 (66.7)	4 (57.1)	27 (93.1)	33 (84.6)
No	1 (33.3)	3 (42.9)	2 (6.9)	6 (15.4)
**Age when first started smoking**				
Number with data (% of those who smoke)	*3 (100)*	*7 (87.5)*	*29 (100)*	*39 (97.5)*
Mean (SD)	15.0 (2.6)	19.4 (4.1)	25.1 (11.0)	23.3 (10.2)
Median (IQR)	16.0 (12.0-17.0)	21.0 (17.0-21.0)	20.0 (18.0-30.0)	20.0 (17.0-25.0)
Min, Max	12.0, 17.0	12.0, 25.0	10.0, 52.0	10.0, 52.0
**Average number smoked per day:**				
**Manufactured cigarettes** Number with data and smoked non-zero value (% of	*3 (100)*	*3 (37.5)*	*12 (41.4)*	*18 (45.0)*

smokers)	4.5 (5.0)	16.4 (14.1)	9.9 (17.3)	10.1 (15.3)
Mean (SD)	3.0 (0.4-10.0)	24.0 (0.1-25.0)	2.0 (1.0-13.0)	2.5 (1.0-20.0)
Median (IQR)	0.4, 10.0	0.1, 25.0	0.1, 60.0	0.1, 60.0
Min, Max				
**Hand-rolled cigarettes**				
Number with data and smoked non-zero value (% of smokers)	*0 (0)*	*1 (12.5)*	*1 (3.4)*	*2 (5.0)*
Mean (SD)	-	12.0 (.)	20.0 (.)	16.0 (5.7)
Median (IQR)	-	12.0 (12.0-12.0)	20.0 (20.0-20.0)	16.0 (12.0-20.0)
Min, Max	-	12.0, 12.0	20.0, 20.0	12.0, 20.0
**Cigars, cheroots, cigarillos**				
Number with data and smoked non-zero value (% of smokers)	*0 (0)*	*0 (0)*	*16 (55.2)*	*16 (40.0)*
Mean (SD)	-	-	3.9 (3.1)	3.9 (3.1)
Median (IQR)	-	-	3.0 (1.5-5.5)	3.0 (1.5-5.5)
Min, Max	-	-	0.4, 10.0	0.4, 10.0
**Number of Shisha sessions**				
Number with data and smoked non-zero value (% of smokers)	*0 (0)*	*0 (0)*	*0 (0)*	*0 (0)*
Mean (SD)	-	-	-	-
Median (IQR)	-	-	-	-
Min, Max	-	-	-	-
**Previously smoked tobacco products, n (%)**				
Number with data	*585 (100)*	*524 (99.4)*	*507 (97.9)*	*1616 (99.1)*
Yes	9 (1.5)	20 (3.8)	46 (9.1)	75 (4.6)
No	576 (98.5)	504 (96.2)	461 (90.9)	1541 (95.4)
**Previously smoked tobacco products daily, n (% of those who previously smoked)**	*9 (100)*	*19 (95.0)*	*46 (100)*	*74 (98.7)*
	4 (44.4)	11 (57.9)	31 (67.4)	46 (62.2)
Number with data	5 (55.6)	8 (42.1)	15 (32.6)	28 (37.8)

Yes<br><br>No				
**Currently uses smokeless tobacco products, n (%)**				
Number with data	*585 (100)*	*523 (99.2)*	*511 (98.6)*	*1619 (99.3)*
Yes	112 (19.1)	52 (9.9)	49 (9.6)	213 (13.2)
No	473 (80.9)	471 (90.1)	462 (90.4)	1406 (86.8)
**Currently uses smokeless tobacco products daily, n (% of those currently using smokeless tobacco)**				
Number with data	*111 (99.1)*	*52 (100)*	*49 (100)*	*212 (99.5)*
Yes	103 (92.8)	35 (67.3)	44 (89.8)	182 (85.8)
No	8 (7.2)	17 (32.7)	5 (10.2)	30 (14.2)

A3 Prevalence of Tobacco Cessation Advice – All Participants

The prevalence of receiving tobacco cessation advice for all participants - both those who do and do not use tobacco - was estimated to be 17.6% (95% CI: 15.7–19.7) in Bangladesh, 22.2% (95% CI: 18.7–26.0) in India, and 15.8% (95% CI: 13.4–18.4) in Pakistan. Table A3a displays the prevalence of tobacco cessation advice overall and stratified by relevant participant characteristics for each country.

Table A3a: Overall and stratified prevalence of being given tobacco cessation advice for all participants, for Bangladesh, India and Pakistan.

	**Bangladesh (n=1346)**		**India (n=501)**		**Pakistan (n=824)**	
**Frequency**<br><br>**Prevalence (95% CI)**	**Number given tobacco cessation advice/ number in group**	**Prevalence % (95% CI)**	**Number given tobacco cessation advice/ number in group**	**Prevalence % (95% CI)**	**Number given tobacco cessation advice/ number in group**	**Prevalence % (95% CI)**
**Overall**	237/1346	17.6 (15.7-19.7)	111/501	22.2 (18.7-26.0)	130/824	15.8 (13.4-18.4)
**Stratified prevalence:**						
**Age**						
18 - 24 years	48/391	12.3 (9.4-15.9)	9/51	17.6 (9.4-30.6)	13/97	13.4 (7.9-21.7)
25 - 39 years	132/661	20.0 (17.1-23.2)	49/226	21.7 (16.8-27.5)	68/389	17.5 (14.0-21.6)
40 - 54 years	53/235	22.6 (17.7-28.3)	37/161	23.0 (17.1-30.1)	32/243	13.2 (9.5-18.0)
55+ years	4/59	6.8 (2.6-16.7)	16/63	25.4 (16.2-37.5)	17/95	17.9 (11.4-26.9)

**Sex**						
Male	227/847	26.8 (23.9-29.9)	104/312	33.3 (28.3-38.8)	121/535	22.6 (19.3-26.4)
Female	10/499	2.0 (1.1-3.7)	7/189	3.7 (1.8-7.6)	9/289	3.1 (1.6-5.9)
**SMI**						
Psychotic disorder	134/835	16.0 (13.7-18.7)	57/286	19.9 (15.7-25.0)	13/77	16.9 (10.1-26.9)
Major depressive disorder with psychotic features	4/69	5.8 (2.2-14.5)	7/32	21.9 (10.8-39.3)	45/385	11.7 (8.8-15.3)
Bipolar disorder (any)	99/442	22.4 (18.8-26.5)	47/183	25.7 (19.9-32.5)	72/362	19.9 (16.1-24.3)
**Duration of the SMI**						
Less than or equal to 2 years	53/382	13.9 (10.8-17.7)	13/84	15.5 (9.2-24.9)	15/168	8.9 (5.5-14.3)
3 to 5 years	77/409	18.8 (15.3-22.9)	19/112	17.0 (11.1-25.1)	33/219	15.1 (10.9-20.4)
6 to 10 years	62/304	20.4 (16.2-25.3)	24/131	18.3 (12.6-25.9)	37/186	19.9 (14.8-26.3)
More than 10 years	44/248	17.7 (13.5-23.0)	52/157	33.1 (26.2-40.8)	45/246	18.3 (13.9-23.6)
**Income tertile**						
Low	104/601	17.3 (14.5-20.5)	54/195	27.7 (21.9-34.4)	61/373	16.4 (12.9-20.5)
Middle	87/430	20.2 (16.7-24.3)	33/165	20.0 (14.6-26.8)	21/194	10.8 (7.2-16.0)
High	45/314	14.3 (10.9-18.7)	22/90	24.4 (16.7-34.4)	48/251	19.1 (14.7-24.5)

Table A3b: Overall and stratified prevalence of being given tobacco cessation advice for those who use tobacco and had a healthcare visit in the past 12-months, for Bangladesh, India and Pakistan.

	**Bangladesh (n=536)**		**India (n=183)**		**Pakistan (n=381)**	
**Frequency Prevalence (95% CI)**	**Number given tobacco cessation advice/ number in group**	**Prevalence % (95% CI)**	**Number given tobacco cessation advice/ number in group**	**Prevalence % (95% CI)**	**Number given tobacco cessation advice/ number in group**	**Prevalence % (95% CI)**

**Overall**	218/536	40.7 (36.6-44.9)	90/183	49.2 (42.0-56.4)	114/381	29.9 (25.5-34.7)
**Stratified prevalence:**						
**Age**						
18 - 24 years	46/110	41.8 (33.0-51.2)	6/17	35.3 (16.8-59.6)	11/30	36.7 (21.6-54.9)
25 - 39 years	118/283	41.7 (36.1-47.5)	39/86	45.3 (35.2-55.9)	61/186	32.8 (26.4-39.9)
40 - 54 years	50/118	42.4 (33.8-51.5)	33/61	54.1 (41.6-66.1)	26/113	23.0 (16.2-31.7)
55+ years	4/25	16.0 (6.1-35.7)	12/19	63.2 (40.3-81.3)	16/52	30.8 (19.8-44.5)
**Sex**						
Male	212/459	46.2 (41.7-50.8)	86/166	51.8 (44.2-59.3)	108/334	32.3 (27.5-37.5)
Female	6/77	7.8 (3.5-16.3)	4/17	23.5 (9.1-48.6)	6/47	12.8 (5.8-25.7)
**SMI**						
Psychotic disorder	125/322	38.8 (33.6-44.3)	47/101	46.5 (37.0-56.3)	11/44	25.0 (14.4-39.8)
Major depressive disorder with psychotic features	4/16	25.0 (9.7-50.9)	5/13	38.5 (17.0-65.7)	38/152	25.0 (18.8-32.5)
Bipolar disorder (any)	89/198	44.9 (38.2-51.9)	38/69	55.1 (43.3-66.3)	65/185	35.1 (28.6-42.3)
**Duration of the SMI**						
Less than or equal to 2 years	50/135	37.0 (29.3-45.5)	8/30	26.7 (13.9-45.0)	12/55	21.8 (12.8-34.6)
3 to 5 years	73/161	45.3 (37.8-53.1)	13/40	32.5 (19.9-48.3)	30/96	31.3 (22.8-41.2)
6 to 10 years	55/133	41.4 (33.3-49.9)	23/46	50.0 (35.9-64.1)	30/93	32.3 (23.6-42.4)
More than 10 years	39/106	36.8 (28.2-46.4)	45/61	73.8 (61.4-83.3)	42/135	31.1 (23.9-39.4)
**Income tertile**						
Low	94/244	38.5 (32.6-44.8)	47/76	61.8 (50.5-72.0)	56/187	29.9 (23.8-36.9)
Middle	79/174	45.4 (38.1-52.9)	23/61	37.7 (26.5-50.4)	18/82	22.0 (14.3-32.2)

High	44/117	37.6 (29.3-46.7)	20/33	60.6 (43.3-75.6)	40/111	36.0 (27.7-45.4)

A4 Association between Smoking Tobacco and Mental and Physical health

Table A4: Descriptive summaries of candidate variables presented by country and whether or not the participant currently smokes tobacco.

	**Bangladesh (n=1500)**		**India (n=1175)**		**Pakistan (n=1304)***	
	**Smoked tobacco (n=408)**	**Does not smoke tobacco (n=1092)**	**Smokes tobacco (n=176)**	**Does not smoke tobacco (n=999)**	**Smokes tobacco (n=315)**	**Does not smoke tobacco (n=989)**
**Depression (PHQ-9 Score)**						
n (%)	408 (100)	1092 (100)	176 (100)	999 (100)	315 (100)	989 (100)
Mean (SD)	10.5 (4.4)	10.8 (4.7)	9.5 (8.1)	5.2 (6.3)	12.4 (6.8)	13.0 (6.8)
Median (IQR)	10.0 (8.0-13.0)	10.0 (8.0-14.0)	8.5 (2.0-16.0)	3.0 (0.0-9.0)	13.0 (7.0-18.0)	13.0 (8.0-18.0)
Min, Max	0.0, 23.0	0.0, 27.0	0.0, 27.0	0.0, 27.0	0.0, 27.0	0.0, 27.0
**PHQ-9 Group, n (%)**						
Number with data						
No/Mild	*408 (100)*	*1092 (100)*	*176 (100)*	*999 (100)*	*315 (100)*	*989 (100)*
depression	174 (42.6)	434 (39.7)	98 (55.7)	791 (79.2)	117 (37.1)	327 (33.1)
Moderate/Severe	234 (57.4)	658 (60.3)	78 (44.3)	208 (20.8)	198 (62.9)	662 (66.9)
depression						
**Anxiety (GAD-7 Score)**						
n (%)	408 (100)	1092 (100)	176 (100)	999 (100)	315 (100)	989 (100)
Mean (SD)	7.9 (3.8)	8.1 (3.9)	7.4 (6.5)	4.1 (5.1)	9.7 (4.8)	10.0 (5.2)
Median (IQR)	8.0 (6.0-11.0)	8.0 (6.0-11.0)	6.0 (1.0-13.0)	2.0 (0.0-6.0)	10.0 (6.0-13.0)	10.0 (6.0-14.0)
Min, Max	0.0, 21.0	0.0, 21.0	0.0, 21.0	0.0, 21.0	0.0, 21.0	0.0, 21.0
**GAD-7 Group, n (%)**	*408 (100)*	*1092 (100)*	*176 (100)*	*999 (100)*	*315 (100)*	*989 (100)*
Number with data	262 (64.2)	716 (65.6)	113 (64.2)	852 (85.3)	157 (49.8)	468 (47.3)
No/Mild anxiety	146 (35.8)	376 (34.4)	63 (35.8)	147 (14.7)	158 (50.2)	521 (52.7)

Moderate/Severe anxiety						
**Diabetes, n (%)**						
Number with data	*395 (96.8)*	*1052 (96.3)*	*149 (84.7)*	*867 (86.8)*	305 (96.8)	970 (98.1)
Diabetes	29 (7.3)	98 (9.3)	23 (15.4)	141 (16.3)	26 (8.5)	95 (9.8)
No diabetes	366 (92.7)	954 (90.7)	126 (84.6)	726 (83.7)	279 (91.5)	875 (90.2)
**Hypertension, n (%)**						
Number with data	*408 (100)*	*1092 (100)*	*176 (100)*	*997 (99.8)*	*315 (100)*	*989 (100)*
Yes	33 (8.1)	110 (10.1)	19 (10.8)	123 (12.3)	73 (23.2)	257 (26.0)
No	375 (91.9)	982 (89.9)	157 (89.2)	874 (87.7)	242 (76.8)	732 (74.0)
**Heart disease, n (%)**						
Number with data	*408 (100)*	*1092 (100)*	*176 (100)*	*999 (100)*	*315 (100)*	*989 (100)*
Yes	2 (0.5)	18 (1.6)	5 (2.8)	28 (2.8)	29 (9.2)	44 (4.4)
No	406 (99.5)	1074 (98.4)	171 (97.2)	971 (97.2)	286 (90.8)	945 (95.6)

*Current smoking status was missing for n=10 participants in Pakistan.

A5 Multiple imputation analysis - Testing the association of determinants of tobacco use

Multiple imputation (MI) analysis was performed as a sensitivity analysis, to compare results under a departure from the MAR assumption that was assumed for the complete-case analysis. For each country separately, an imputation model containing the outcome variable, covariates and auxiliary variables was used to impute missing data using chained equations, for m=10 imputed datasets. Next, the same model as used in the main analysis was performed for each of the imputed datasets, for each country. Finally, the results from each of the m=10 models were combined using Rubin's rules (Rubin, 1987). Parameter estimates are presented in Table A5 for Bangladesh, India and Pakistan, respectively. Estimates are reported alongside the complete-case analysis for comparison.

Table A5: Association of mental and physical health conditions with tobacco use. Parameter estimates from a logistic regression model with multiple imputation are compared to a complete-case analysis.

	**Complete-case analysis (n=3575)**		**Multiple Imputation analysis (n=3989)**	
**Variable**	**OR (95% CI)**	**p-value**	**OR (95% CI)**	**p-value**
**Sex**				

Male	Reference	Reference	Reference	Reference
Female	0.14 (0.12-0.17)	p<0.01	0.14 (0.12-0.17)	p<0.01
**Age group**				
18-24 years	Reference	Reference	Reference	Reference
25-39 years	1.90 (1.51-2.39)	p<0.01	1.89 (1.52-2.36)	p<0.01
40-54 years	2.58 (1.97-3.37)	p<0.01	2.57 (1.99-3.32)	p<0.01
55+ years	2.20 (1.54-3.15)	p<0.01	2.18 (1.55-3.08)	p<0.01
**SMI diagnosis**				
Bipolar disorder	Reference	Reference	Reference	Reference
Major depressive disorder with psychotic features	0.66 (0.52-0.85)	p<0.01	0.67 (0.53-0.85)	p<0.01
Psychotic disorder	0.94 (0.79-1.13)	p=0.504	0.95 (0.80-1.13)	p=0.586
**SMI duration**				
<2 years	Reference	Reference	Reference	Reference
3-5 years	1.16 (0.93-1.45)	p=0.186	1.12 (0.91-1.39)	p=0.288
6-10 years	1.25 (0.99-1.58)	p=0.062	1.22 (0.98-1.53)	p=0.077
>10 years	1.12 (0.89-1.43)	p=0.333	1.08 (0.86-1.35)	p=0.526
**Level of education**				
No formal education	Reference	Reference	Reference	Reference
Primary education	0.62 (0.48-0.79)	p<0.01	0.59 (0.47-0.76)	p<0.01
Secondary education	0.49 (0.37-0.66)	p<0.01	0.50 (0.38-0.65)	p<0.01

Higher/more than secondary	0.37 (0.28-0.48)	p<0.01	0.36 (0.28-0.47)	p<0.01
**Income tertile**				
Low	Reference	Reference	Reference	Reference
Middle	0.87 (0.73-1.05)	p=0.143	0.89 (0.75-1.06)	p=0.179
High	0.98 (0.81-1.19)	p=0.838	0.99 (0.82-1.20)	p=0.957
**Depression**				
No/Mild depression	Reference	Reference	Reference	Reference
Moderate/Severe depression	1.18 (0.99-1.42)	p=0.071	1.20 (1.01-1.43)	p=0.040
**Anxiety**				
No/Mild anxiety	Reference	Reference	Reference	Reference
Moderate/Severe anxiety	1.34 (1.12-1.61)	p<0.01	1.38 (1.16-1.65)	p<0.01
**Type 2 Diabetes**				
No Diabetes	Reference	Reference	Reference	Reference
Diabetes (HbA1c ≥ 6.5)	1.06 (0.82-1.37)	p=0.669	1.04 (0.81-1.35)	p=0.747
**Hypertension/high blood pressure (BP ≥ 140/90)**				
No	Reference	Reference	Reference	Reference
Yes	0.93 (0.75-1.15)	p=0.502	0.98 (0.80-1.21)	p=0.851
**Heart disease**				
No	Reference	Reference	Reference	Reference
Yes	1.16 (0.76-1.76)	p=0.492	1.15 (0.77-1.73)	p=0.500
**Country**				

Bangladesh	Reference	Reference	Reference	Reference
India	0.51 (0.41-0.63)	p<0.01	0.51 (0.42-0.63)	p<0.01
Pakistan	1.08 (0.87-1.34)	p=0.493	1.05 (0.85-1.30)	p=0.650

OR, Odds ratio. CI, Confidence interval.

© 2023 Rajan S. et al.
